# Effect of Sc on the Hot Cracking Properties of 7xxx Aluminum Alloy and the Microstructure of Squeeze Castings

**DOI:** 10.3390/ma14226881

**Published:** 2021-11-15

**Authors:** Yongtao Xu, Zhifeng Zhang, Zhihua Gao, Yuelong Bai, Purui Zhao, Weimin Mao

**Affiliations:** 1 National Engineering & Technology Research Center for Non-Ferrous Metals Composites, GRINM Group Corporation Limited, Beijing 101407, China; xyt93upc@hotmail.com (Y.X.); bai_yuelong@163.com (Y.B.); zpr@mail.nwpu.edu.cn (P.Z.); 2 School of Materials Science and Engineering, University of Science and Technology Beijing, Beijing 100088, China; mao_wm@ustb.edu.cn; 3 Grinm Metal Composites Technology Co., Ltd., Beijing 101407, China; 4 General Research Institute for Nonferrous Metals, Beijing 100088, China; 5 GRIMAT Engineering Institute Co., Ltd., Beijing 101407, China

**Keywords:** aluminum alloys, hot tearing, microstructure, mechanical properties

## Abstract

In this paper, the effect of adding the refiner Sc to the high Zn/Mg ratio 7xxx series aluminum alloy melt on the hot tearing performance, microstructure, and mechanical properties of the alloy is studied. The hot tearing performance test (CRC) method is used to evaluate the hot tearing performance of the alloy. The squeeze casting process was used to form solid cylindrical parts to analyze the structure and properties of the alloy. This study shows that the hot cracking sensitivity of the alloy after the addition of the refiner Sc is significantly reduced. The ingot grain size is significantly reduced, and the average grain size is reduced from about 86 μm to about 53 μm. While the mechanical properties are significantly improved, and the tensile strength reduced from 552 MPa is increased to 571 MPa, and the elongation rate is increased from 11% to 14%.

## 1. Introduction

The high-end manufacturing fields such as aerospace, rail transit, national defense, and military industry have increasingly urgent demands for large, complex, high-strength, and tough aluminum alloy structural parts. The research on high-strength and tough cast aluminum alloy materials has attracted much attention [1,2,3,4]. At present, the high strength and toughness 7xxx series alloys are mainly deformed aluminum alloys. After deformation processing and heat treatment, the material strength reaches or exceeds the performance of most low-carbon steel and cast steel materials. It has been widely used in lightweight parts in many special conditions [5]. However, this kind of high strength and toughness aluminum alloy usually has a high degree of alloying, wide solidification temperature range, large solidification shrinkage, high alloy hot cracking tendency, poor casting performance, and direct casting molding is very difficult [6]. The castings also have coarse grains and segregation [7]. These serious problems make it difficult for the mechanical properties of the material to reach the level of deformed aluminum alloy. Therefore, the research and development of Al-Zn-Mg-Cu that high-strength and toughness cast aluminum alloys with high mechanical properties and good casting properties to meet the urgent needs of lightweight and low-cost key structural components of aerospace, rail transit, and national defense and military industries, and to realize equipment upgrading is of great significance [8,9,10].

Due to the wide solidification temperature range of 7xxx series aluminum alloys, when crystallized under pressure, the molten metal-rich in alloying elements that are not solidified between the growing dendrites will be forcibly squeezed out and concentrated to the hot joints of the casting, forming abnormal segregation and affecting the uniformity of the casting and the larger internal stress. In addition, the grain size of the 7xxx series aluminum alloy has a very important influence on the mechanical properties and hot tearing properties of the alloy [11,12]. Since 7xxx series aluminum alloys have relatively few eutectic components that have a feeding effect during solidification, it is difficult to flow when blocked by dendrites, and hot tearing is easily generated. The smaller the grain size of the alloy, the better the fluidity of the alloy, and the better the shrinkage during cooling and solidification. At the same time, the smaller grain size will help improve the tensile strength and ductility of the alloy. To obtain a fine-grained structure, it is necessary to control the solidification conditions to increase the number of crystal nucleation cores while suppressing grain growth. Therefore, adding a refiner to the melt can effectively refine the grain structure of the alloy [13], which is considered the simplest and most effective method. Sc and Zr elements are commonly used grain refiners in 7xxx aluminum alloys [14,15,16,17,18]. When a small amount of Zr is added to the melt, the clustered atomic groups in the aluminum alloy melt interact with the Zr elements to form atomic clusters. These atomic clusters eventually develop into the nucleation core of new grains. With the increase of the content of Zr element, the peritectic reaction will occur in the aluminum alloy and become the core of the equilibrium grains, thereby inhibiting the growth of grains. However, the solubility of Zr element in the melt is relatively small, and the effect of crystal grain refinement in the actual non-equilibrium solidification process is limited. Dev [11] research found that adding 0.1% of Zr to commercial aluminum alloys and adding Sc content at least higher than 0.18% can obtain a non-dendritic structure and significantly refine the grains. The addition of Sc and Zr into Al alloys results in the formation of a complex Al3(Sc, Zr) phase [19,20,21]. While the content of the Sc element alone is more than 0.55% to observe the significant grain refinement effect. In the actual production process, the total amount of Sc and Zr is generally within 0.3% when the compound is added, which can not only achieve the obvious refinement effect but also minimize the cost. Although many studies have been carried out on the microstructure and properties of 7xxx aluminum alloys by adding Sc and Zr, there is no report on the effects of adding Sc on the casting properties of new high-strength and tough cast aluminum alloys with a high Zn/Mg ratio.

Therefore, this paper studies the effect of Sc on the hot tearing performance and squeeze casting microstructure properties of Al-7Zn-1.75Mg-1.3Cu-0.15Zr alloy with a high Zn/Mg ratio (Zn/Mg = 4) and controls the total amount of Sc and Zr to be within 0.3%, and clarifies that the addition of Sc element on the alloy heat, the mechanism of cracking performance, microstructure and mechanical properties is expected to provide a theoretical basis for the optimal design of high strength and toughness 7xxx series cast aluminum alloy composition.

## 2. Experimental

### 2.1. Alloy Preparation

The experiment uses self-made 7xxx series aluminum alloy with a high Zn/Mg ratio. The experimental raw materials are industrial high-purity aluminum (99.99%), pure zinc (99.92%), pure magnesium (99.95%), Al-50Cu, Al-5Zr, and Al-2Sc. For the master alloy, the chemical composition of the alloy measured in the experiment is shown in Table 1. The liquidus temperature of the alloy is calculated to be 632 °C by thermodynamic analysis. During smelting, a well-type resistance furnace is used, and a rotating argon gas injection is used for degassing and slagging treatment, and the alloy melt is kept in a heat preservation state to prepare for the experiment.

### 2.2. Experimental Device for a Thermal Cracking Performance

**Figure 1:**
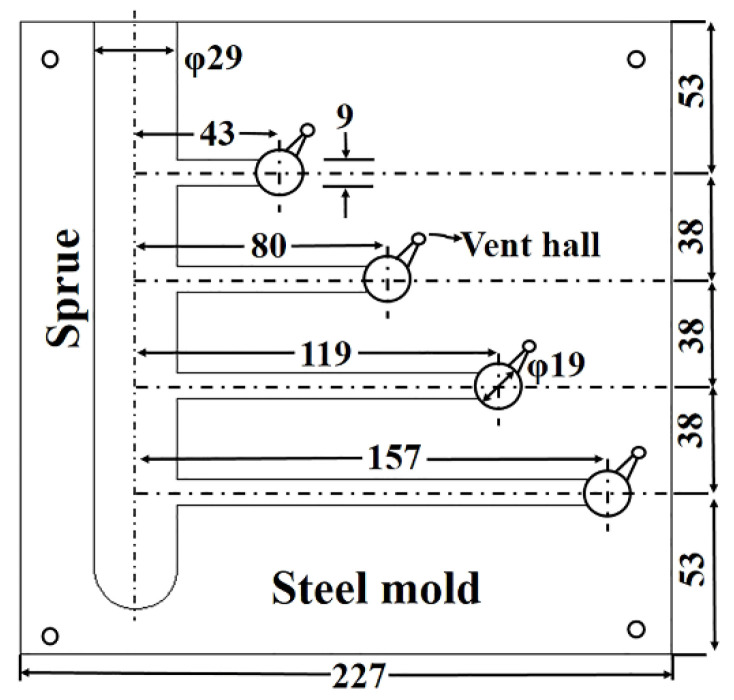
Shrink rod mold for a hot tearing experiment.

The hot tearing performance test (CRC) mold used in the experiment is shown in Figure 1 below. The runner diameter of the mold is 29 mm. There are four hot tearing rods with a diameter of 9 mm distributed on the runner, with lengths of 43 mm, 80 mm, 119 mm, and 157 mm, respectively. There is a shrinking ball with a diameter of 19 mm at the top of the rod to limit the shrinkage after the melt is solidified. The preheating temperature of the CRC mold is 250 °C, the surface is sprayed with BN coating, the melt casting temperature is 720 °C, and the HTS value is calculated using Argo [22] calculation formula:

$$\mathrm{HTS}=\sum \left(f_{crack}f_{length}f_{location}\right)$$

Among them, $f_{crack}f_{length}$ and $f_{location}$, respectively, represent the size of the crack, the length of the test bar where the crack occurs, and the location where the crack occurs on the test bar. When the value of $f_{crack}$ is 1, the crack is hairline, 2 represents the crack is hairline, and 3 represents the crack for severe cracks, 4 represents complete fracture; $f_{length}$ value 4 represents the longest hot tearing rod, 8 represents the second-longest hot tearing rod, 16 represents the second shortest hot tearing rod, and 32 represents the shortest hot tearing rod; $f_{location}$ value 1 represents that the fracture occurred at the gate, a value of 2 represents that it occurred at the constrained ball position, and a value of 3 represents that the fracture occurred at the middle of the hot tearing rod.

### 2.3. Organization and Performance Analysis

**Figure 2:**
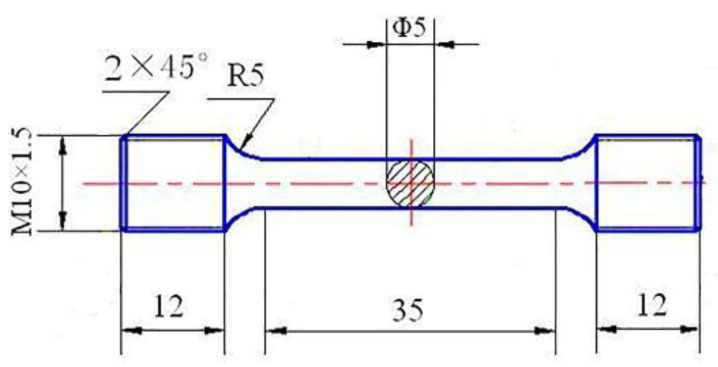
Sample geometry of a tensile specimen (unit: mm).

After degassing and slagging, the alloy is poured into a stainless steel crucible with a preheating temperature of 350 °C. The crucible size is φ 70 mm × H 180 mm. When the melt temperature drops to 660 °C, it is cast into a die forging machine and squeezed into a solid cylindrical ingot. The size of the cylindrical ingot is φ 60 mm × H 90 mm, the mold temperature is 150 °C, the molding pressure is 100 Mpa, and the pressure holding time is 20 s. Cut the solid cylindrical ingot along the radial direction and cut the metallographic sample with a size of 25 mm × 10 mm × 6 mm. After polishing and anodic coating, observe the alloy microstructure with a ZEISS Aovert 200 MAT optical microscope. Analyze the grain size of the alloy and observe the morphology of the grains in the alloy by using the cut-line method. The coating liquid is 2.5% HBF4 solution. The coating voltage is 30 V, the coating current is controlled below 1 mA, and the coating time is 60 s. Another sample was taken for 450 °C/3 h + 460 °C/3 h + 470 °C/3 h solid solution and 120 °C/24 h aging heat treatment. Analyze the mechanical properties of the sample. Tensile properties were performed on a CSS-44100 electronic universal testing machine with a 2 mm/min loading speed. The yield strength of the material was identified at 0.2% plastic strain. A minimum of three mechanical tests was performed on each sample, and their statistical scatter determined the measurement errors. The tensile specimens are illustrated in Figure 2. The fracture morphology of the alloy was characterized by SEM and analyzed by the alloy fracture mode.

## 3. Results

### 3.1. Results of Alloy Microstructure

**Figure 3:**
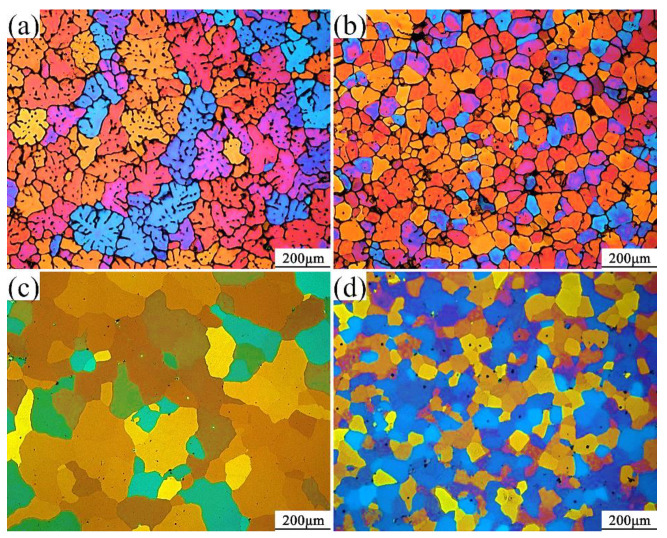
The microstructure of the alloy squeeze casting into a solid cylindrical part under different conditions: (**a**) as-cast, no Sc; (**b**) as-cast, 0.15% Sc; (**c**) heat-treated state, no Sc; (**d**) heat-treated state, 0.15% Sc.

**Figure 4:**
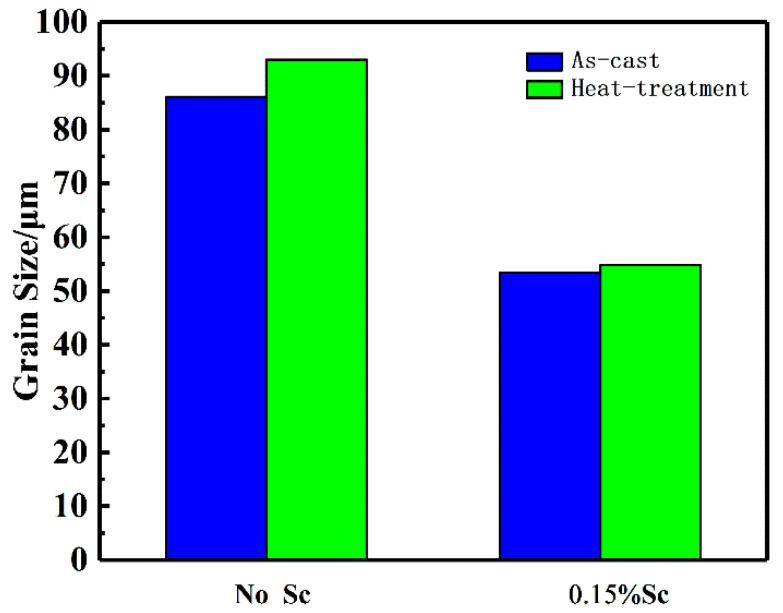
The average grain size of the alloy in different conditions.

Figure 3 shows the as-cast and heat-treated microstructures of the alloy squeeze casting into a solid round casting with or without the addition of Sc. It can be seen from Figure 3a that as a result of solid round castings, the morphology of the ingot grains is very irregular, most of which are coarse dendritic structures of the alloy without the addition of Sc. Most of the grains are distributed in size between 100 and 150 μm. The average grain size calculated by the tangent method is about 86 μm. After heat treatment, as shown in Figure 3c, the grain boundary eutectic phase in the alloy disappears and is completely dissolved into the matrix. Most of the grains have obvious mergers and growing phenomenon. Dendrites disappear, and the spheroidization of crystal grains is obvious. The size of most grains is distributed between 200 and 300 μm. The average grain size calculated by the tangent method is about 93 μm. A small amount of small-size crystal grains have spheroidization. After adding 0.15% of the Sc element, as shown in Figure 3b, the grain size is significantly reduced, and the grain size is mostly distributed between 50 and 100 μm. The average grain size is 53 μm, and the grain morphology is not regular equiaxed crystals, and a very small amount of dendrites and rose crystals can be observed at the same time. After heat treatment, the grain boundary eutectic phase in the alloy is completely dissolved into the inside of the matrix, and a small number of crystal grains are left with massive Al3(Sc, Zr) phase. The average grain size calculated by the tangent method is about 55 μm. The grain size has almost no change, and the grain morphology almost completely becomes equiaxed spherical crystals. The average grain size of the alloy in different states is shown in Figure 4.

### 3.2. Thermal Cracking Performance Results of the Alloy

**Figure 5:**
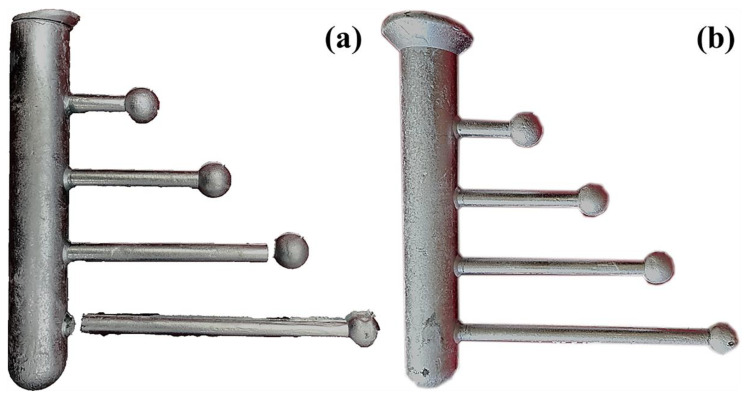
Hot tearing performance samples under treatment with or without the addition of Sc element: (**a**) No Sc; (**b**) 0.15% Sc.

**Figure 6:**
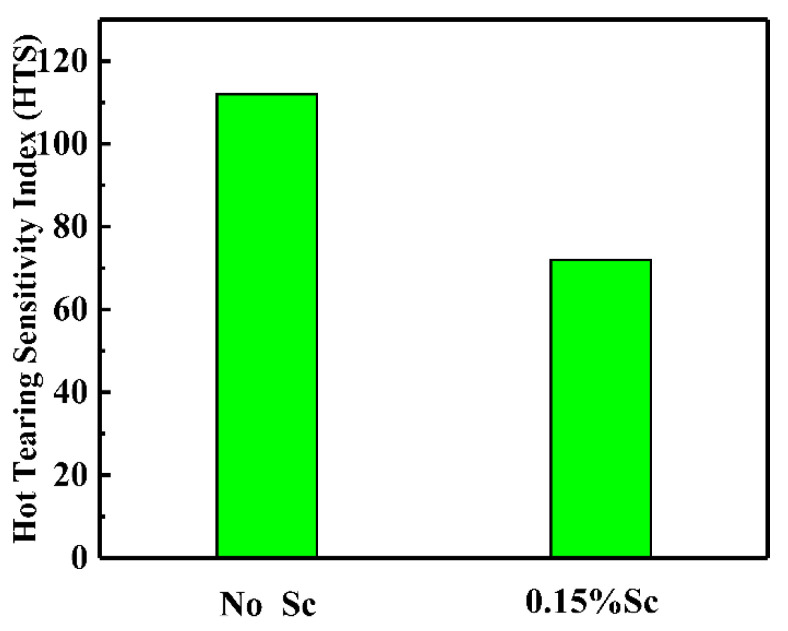
Alloy hot tearing sensitivity value with or without the addition of Sc treatment.

Figure 5 shows the hot tearing performance sample under the condition of adding Sc treatment, and Figure 6 shows the evaluation result of the alloy hot tearing sensitivity value calculated by the Argo formula under the condition of adding Sc treatment. The fractures of different sizes and distributions can be seen under the processing conditions, and the fractures are mainly distributed at the gate opening. There is almost no hot tearing in the middle of the hot cracking rod. Comparing the hot tearing rods of the alloy under the two conditions, after adding 0.15% of the Sc element, the probability of cracks on the hot tearing rod becomes smaller, and the crack size is also significantly reduced. The value calculated by the Argo formula shows that after adding the Sc element, the hot tearing sensitivity index of the alloy is significantly reduced.

### 3.3. Results of Alloy Mechanical Properties

**Figure 7:**
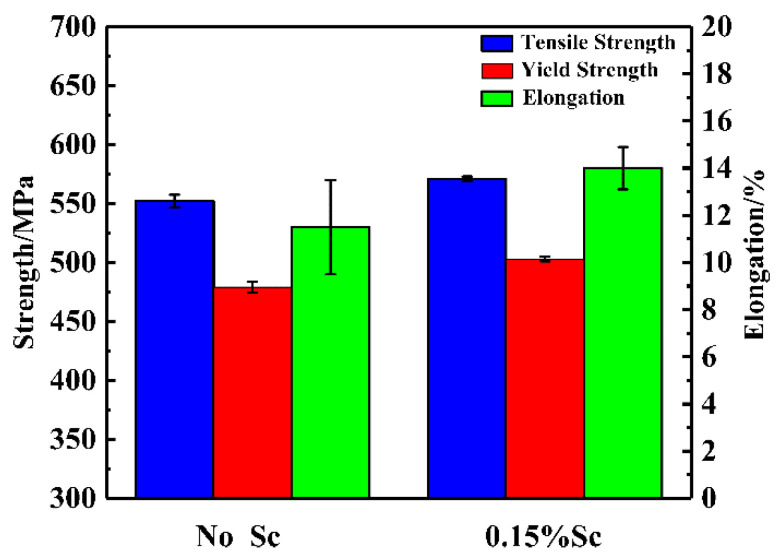
Mechanical properties of alloys under different conditions.

Figure 7 shows the tensile mechanical properties of the alloy at room temperature with or without the addition of the Sc element. By comparison, after heat treatment, the alloy’s tensile strength that without no Sc is 552 MPa, the elongation is about 11%, and the elongation error is relatively large. After adding 0.15% of the Sc element, the tensile strength and elongation of the alloy are significantly improved. The tensile strength is about 571 MPa, the elongation is increased to about 14%, and the yield strength is also increased to more than 500 MPa.

## 4. Analysis and Discussion

### 4.1. Analysis of Alloy Microstructure

As an important microalloying element in high-strength and toughness aluminum alloys, the Zr element can significantly refine the alloy grain size. From the aluminum-rich end of the Al-Zr phase diagram, it can be seen that when the Zr content is greater than 0.11 wt%, the melt will preferentially form the Al3Zr phase during the cooling process. As the temperature drops to 660 °C, aluminum atoms will attach to the primary Al3Zr. The Peritectic reaction occurs on the phase [23]:

L + Al3Zr → α-Al

When Al3Zr exists in the melt as a finely dispersed primary phase, it can be used as the core of α-Al nucleation. When the grains are refined, the non-equilibrium eutectic phase during solidification becomes dispersed, and its quantity and distribution are changed. At the same time, the shrinkage stress and the hot tearing sensitivity of the alloy during solidification are reduced. Although the Zr element can refine crystal grains, its refinement effect is limited. Since crystal formation and growth occur almost simultaneously when the solidification process of the alloy is improperly controlled. The Al3Zr phase as the core of the nucleation will gradually grow into a coarse primary Al3Zr phase, making it difficult for the refinement effect to be exerted to the best extent. To achieving the purpose of grain refinement, a large amount of zirconium-containing master alloys have to be added, which not only causes the purity of the melt to decrease but also causes the formation of coarse primary Al3Zr phases more easily. When the solid round ingot is formed by squeeze casting in this experiment without the Sc element and the additional amount of Zr element is less, as the melt is cooled and solidified, the content of nucleation point Al3Zr in the melt is less, which has a limiting effect on grain refinement. The melt then undergoes non-equilibrium solidification, and a large number of dendrites growing into the liquid are generated at the front of the solidification interface. As the temperature continues to decrease, the dendrites will continue to grow. After heat treatment, the grain boundary eutectic phase is completely dissolved into the inside of the matrix. The crystal grains are in contact with each other, and the phenomenon of merging and growing occurs as the temperature rises.

The Sc element is the most effective grain refiner. The grain refinement effect caused by the addition of Sc mainly comes from the Al3Sc particles precipitated during the melt cooling process. Because the Al3Sc phase in structure and size with the aluminum alloy matrix crystal point is similar to the matrix, when the Al3Sc phase is resolved from the supersaturated solid solution, high-density and stable spherical particles coherent with the matrix can be formed, which can serve as a potential nucleation site for α-Al. There is mainly liquid at the aluminum-rich end of the Al-Sc phase diagram. The α-Al and Al3Sc act crystal reaction, and the grain refinement effect can only work when the Sc content exceeds the eutectic component (0.55%). However, when the Sc content is too high, the non-equilibrium supersaturated Sc solid solution will agglomerate when it is separated so that the beneficial effect of Sc addition on the aluminum alloy will disappear. Therefore, the Sc addition should generally not exceed 0.35%. However, the refinement effect on the alloy is weakened. Therefore, a small amount of Zr is added together when adding Sc, so that a large number of high melting point heterogeneous nucleation particles Al3(Sc, Zr) particles will be generated in the melt. The particles are similar in structure and size to the aluminum matrix crystal lattice. They can be used as potential nucleation sites of aluminum crystal grains. As the temperature decreases, aluminum atoms in the melt will attach to the primary Al3(Sc, Zr) particles to undergo a peritectic reaction to achieve the effect of grain refinement. The heat treatment process can minimize particle agglomeration sensitivity. In this experiment, when the Zr content is 0.14%, and 0.15% Sc is added, the grain size is significantly refined. The average grain size reduced from 86μm without Sc to 53μm after adding Sc. The crystallization phenomenon disappeared, and the morphology of the crystal grain changed from a coarse dendritic structure to an irregular spherical crystal. After heat treatment, the grain boundary eutectic phase is completely dissolved into the inside of the matrix, and the grain size tends to increase. However, the phenomenon of growth is not obvious.

### 4.2. Analysis of Alloy Thermal Cracking Performance

When the alloy composition is the same or similar, the hot tearing sensitivity of the alloy is greatly affected by the grain size and grain morphology. The grain refinement of the alloy improves the flow of the melt between the dendrites due to the deterioration of the crystal grain and reduces the size of the grain boundary eutectic phase between the crystal grains. Under the conditions of certain grain boundary eutectic phase content, alloys with refined grains are prone to produce a large number of, but small, grain boundaries, while alloys with coarse grains are more likely to form a small number of, but large, grain boundary eutectic phases. The latter is undoubtedly more harmful to the hot tearing resistance of the alloy. At the same time, grain refinement can reduce the effective solidification range of the alloy and improve the strain tolerance of the alloy. The strain distribution of the alloy during solidification is not uniform and will concentrate at the grain boundaries. The fine grain structure contains more grain boundaries, so it can better withstand the thermal strain and reduce the degree of strain concentration, thereby reducing the thermal strain during solidification and improving the alloy’s resistance to thermal cracking sensitivity [24].

In this experiment, when the refiner Sc element is not added to the alloy, the alloy is mainly composed of coarse, uneven, and optimally oriented columnar crystals. Near the end of solidification, solid dendrites begin to bridge. At this time, the fluidity of the mushy zone decreases, and the ability of the remaining liquid between the grain boundaries to feed is reduced, unable to overcome the tensile strain, and eventually lead to easy cracking in the mushy zone. After adding the Sc element, the solidified structure of the alloy becomes fine equiaxed grains. The fine equiaxed grain structure replaces the coarse columnar grain structure, and the change of grain size and morphology reduces the strain and rate of strain of the mushy zone during alloy solidification, which greatly reduces the hot cracking tendency of the alloy. At the same time, compared with the coarse columnar crystals, the fine equiaxed crystal structure has more tortuous, more uniform distribution and fine grain boundaries, which makes the crack propagation more difficult, and the hot tearing sensitivity of the alloy will be reduced significantly.

### 4.3. Analysis of Alloy Mechanical Properties

The mechanical properties of aluminum alloy were improved effectively by adding Sc. The primary Al3(Sc, Zr) particles formed in the casting process can refine the grain, while the fine dispersive secondary Al3(Sc, Zr) particles formed in the heat treatment process can stabilize the substructure and further improve the strength of the alloy. Since the Sc and Zr elements added to the aluminum alloy mainly precipitate in the form of Al3(Sc, Zr) particles during the heat treatment process, and the content of Sc and Zr in the matrix is very small, the aging precipitation kinetics of the matrix alloy will not be affected, and the precipitation of the strengthening phase η’(MgZn2) in the alloy will not be affected. Therefore, the increase of alloy strength is mainly related to fine grain strengthening and Al3(Sc, Zr) precipitation strengthening. After the addition of the Sc element, the grain boundaries are increased, and there are more impurity atoms near the grain boundaries. At the same time, the grain orientations on both sides of the grain boundaries are different, which increases the dislocation slip resistance near the grain boundaries. After heat treatment, due to the higher amount of refiner added in the grain, the capacity of the precipitated Al3(Sc, Zr) phase to hinder the passage of dislocation increases, which increases the strength of the alloy.

**Figure 8:**
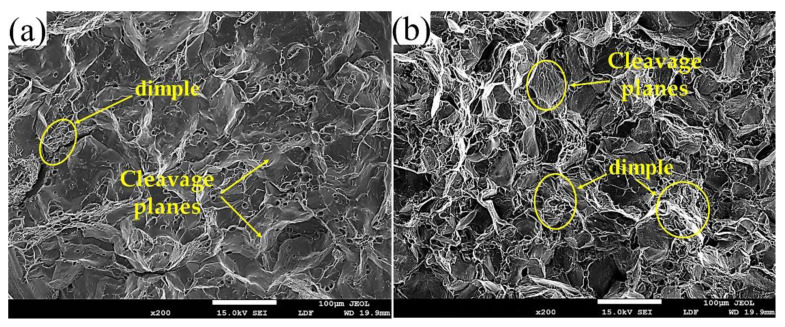
Tensile fracture morphology of the alloy under different conditions: (**a**) Without Sc; (**b**) With Sc.

Figure 8 shows the tensile fracture morphology of the two alloys obtained by SEM at room temperature. From Figure 8a, when the refiner Sc element is not added, the tensile fracture of the alloy exhibits a larger intergranular toughness. There are a large number of lamellar cleavage planes on the fracture surface and a small number of small-sized dimple pits on the cleavage surface. This indicates that the alloy fractures during shearing and mixed fracture along with the crystal dimples. On the one hand, due to the crystal, the grain size is larger and the grain boundaries are relatively flat [25,26,27]. The coarse precipitation phase precipitated at the grain boundaries reduces the strength of the grain boundaries. So, it is conducive to the concentration of stress and strain, and promotes the generation of cracks in the grain boundary precipitation phase and the expansion of the grain boundaries. On the other hand, in some specific areas inside the coarse grains, dislocations will move along a certain crystal plane, causing a large number of dislocations to form shear bands in this slip plane. Eventually, cracks and transgranular shear fractures will occur. It can be seen from Figure 8b that when the Sc refiner is added to the alloy, the grain size is further refined, and the fracture surface is completely composed of a large number of equiaxed small dimples and a small number of cleavage surfaces. It shows a high toughness fracture, which is consistent with the performance of the elongation after fracture in the tensile test. Due to the small grain size, many grain boundaries, and twists, the crystal grains are tightly combined. When cracks occur at the grain boundary precipitation phase and the second phase in the grain, the cracks will be connected in series to cause a transcrystalline dimple fracture [28]. If cracks are generated in the shear zone, the shear fracture will occur. In addition, there are fewer shear fractures, and transgranular dimple fractures dominate. It is relatively difficult for dislocations to grow in grain-refined alloys. The plasticity of the alloy improves, and the elongation increases.

## 5. Conclusions

1. When the alloy is not added with the Sc element, the grain structure of the squeeze casting is coarse dendrites, with an average grain size of about 86 μm. After adding 0.15% of the Sc element, the grain size of the casting is significantly refined, and the average grain size is about 53 μm. The morphology of the crystal grains changed from coarse dendritic crystals to equiaxed crystals.
2. When the Sc element is not added, the alloy is prone to hot cracking. After adding 0.15% of the Sc element, the fluidity of the alloy is improved, the hot tearing sensitivity is significantly reduced, and the hot tearing sensitivity index drops from 120 to about 80.
3. When the Sc element is not added, the mechanical properties of the squeeze castings are poor. The tensile strength is 552 MPa, and the elongation is about 11%. After adding 0.15% of the Sc element, the tensile strength of the alloy is increased to 571 Mpa, and the elongation is increased to 14%. The alloy fracture mode changed from mixed fracture without adding the Sc element to fracture dominated by ductile fracture after adding the Sc element.

## Figures and Tables

**Table 1:** Chemical composition of the experimental alloy (wt %).

Zn	Mg	Cu	Zr	Sc	Fe	Si	Others	Al
7.15	1.79	1.28	0.14	-	0.002	0.002	≤0.01	Bal.
7.08	1.76	1.3	0.14	0.15	0.002	0.002	≤0.01	Bal.

## Data Availability

Date is contained within the article.
